# Indicators of AEI Applied to the Delaware Estuary

**DOI:** 10.1100/tsw.2002.346

**Published:** 2002-04-17

**Authors:** Lawrence W. Barnthouse, Douglas G. Heimbuch, Vaughn C. Anthony, Ray W. Hilborn, Ransom A. Myers

**Affiliations:** LWB Environmental Services, Inc.,105 Wesley Lane, Oak Ridge, TN 37830, USA; PBSJ, 12101 Indian Creek Court, Beltsville, MD 20705, USA; P.O. Box 459, Gaecklin Rd., Boothbay, ME 04537, USA; University of Washington, School of Fisheries, Box 355020, Seattle, WA 98195, USA; 5Dalhousie University, Department of Biology, Halifax Nova Scotia, Canada B3H 4J1,

**Keywords:** 316(b), adverse environmental impact, AEI, biological indicators, fish populations

## Abstract

We evaluated the impacts of entrainment and impingement at the Salem Generating Station on fish populations and communities in the Delaware Estuary. In the absence of an agreed-upon regulatory definition of “adverse environmental impact” (AEI), we developed three independent benchmarks of AEI based on observed or predicted changes that could threaten the sustainability of a population or the integrity of a community.

Our benchmarks of AEI included: (1) disruption of the balanced indigenous community of fish in the vicinity of Salem (the “BIC” analysis); (2) a continued downward trend in the abundance of one or more susceptible fish species (the “Trends” analysis); and (3) occurrence of entrainment/impingement mortality sufficient, in combination with fishing mortality, to jeopardize the future sustainability of one or more populations (the “Stock Jeopardy” analysis).

The BIC analysis utilized nearly 30 years of species presence/absence data collected in the immediate vicinity of Salem. The Trends analysis examined three independent data sets that document trends in the abundance of juvenile fish throughout the estuary over the past 20 years. The Stock Jeopardy analysis used two different assessment models to quantify potential long-term impacts of entrainment and impingement on susceptible fish populations. For one of these models, the compensatory capacities of the modeled species were quantified through meta-analysis of spawner-recruit data available for several hundred fish stocks.

All three analyses indicated that the fish populations and communities of the Delaware Estuary are healthy and show no evidence of an adverse impact due to Salem. Although the specific models and analyses used at Salem are not applicable to every facility, we believe that a weight of evidence approach that evaluates multiple benchmarks of AEI using both retrospective and predictive methods is the best approach for assessing entrainment and impingement impacts at existing facilities.

## INTRODUCTION

Section 316(b) of the Clean Water Act requires that "the location, design, construction, and capacity of cooling water intake structures reflect the best technology available for minimizing adverse environmental impact." However, neither the act itself nor any applicable regulatory guidance provides an explicit definition of the term "adverse environmental impact" (AEI) or explicit criteria for determining when an AEI has occurred or might potentially occur.

The draft Section 316(b) guidelines[1], which were never formally approved, contain language suggesting that the focus of AEI determinations should be on the impairment of populations and communities:

> *"Adverse environmental impacts occur when the ecological function of the organism(s) of concern is impaired or reduced to the level which precludes maintenance of existing populations; a reduction in optimum sustained yield to sport and/or commercial fisheries results; threatened or endangered species of aquatic life are directly or indirectly involved; and/or the magnitude of the existing or proposed damage constitutes an unmitigatable loss to the aquatic system."*

Because all organisms have finite life spans, the reproducing population is the smallest ecological unit that is persistent in time. Assessments of the impacts of entrainment (defined as the transport of fish eggs or larvae and other small aquatic organisms through a cooling-water system) and impingement (defined as the trapping of fish on the screens that prevent large debris from being drawn into a cooling-water systems) at Hudson River power plants, which were the focus of intensive study during the 1960s and 1970s, focused on

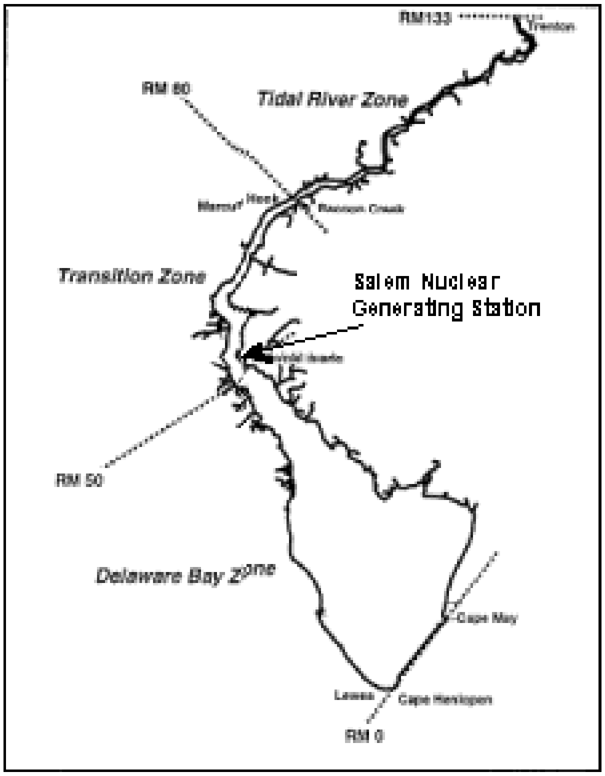

**FIGURE 1.** Longitudinal zones of the Delaware Estuary.

potential reductions in the abundance and yield of fish populations[2,3]. In this and other studies, assessments relied on concepts and methods that are ultimately grounded in resource management, especially fisheries science.

We designed an impact assessment approach for the Salem Generating Station, located in Lower Alloways Creek, NJ, on the Delaware Estuary (Fig.1), based on these precedents and on more recent guidance from the U.S. Environmental Protection Agency (EPA) on the use of multiple lines of evidence in ecological risk assessments. Our approach was intended to address impacts of Salem on the balance of the fish community present in the Delaware Estuary and on the sustainability of specific representative fish populations that utilize the estuary. Salem began commercial operation in 1977 and, except for outages for maintenance, refueling, and system upgrades, has operated continuously since that time. Because of its size, location, and cooling-water withdrawal rate, Salem has long been identified as a source of potential intake structure-related impacts, and was the subject of an extensive Section 316(b) Demonstration in 1984, which was updated in 1991 and 1993. All of these documents relied heavily on assessment approaches, especially the use of population-level assessment models, which were previously used in the Hudson River assessment studies. The assessment described in this paper was prepared to support a New Jersey Pollutant Discharge Elimination System Permit Renewal Application submitted by Public Service Electric and Gas (PSEG) in 1999.

The Delaware Estuary extends 133 mi (214 km) from the head of tide at Trenton, NJ, to the mouth of Delaware Bay. It is one of the largest estuaries on the Atlantic Coast, with an open-water area of approximately 725 $mi^2$, not including tributaries and fringing marsh-plain areas. The estuary is divided into three longitudinal zones (Fig. 1) based on salinity, turbidity, and biological productivity: the Tidal River Zone, a zone of low salinity, low turbidity, and moderate biological productivity extending from the head-of-tide at Trenton, NJ (RM 133), to Marcus Hook, PA (RM 80); the Transition Zone, a zone of high turbidity, variable salinity, and variable biological productivity extending from Marcus Hook (RM 80) to Artificial Island (RM 50); and the Delaware Bay Zone, a zone of high salinity, low turbidity, and high biological productivity extending from Artificial Island to the mouth of the bay.

Salem has been operating for more than 20 years. During this period an enormous quantity of data has been collected concerning environmental conditions in the Delaware Estuary and concerning the status and biological characteristics of fish populations that utilize the estuary. We used all available and relevant data for the assessment, including in-plant sampling data, ichthyoplankton and finfish surveys, and coastwide stock assessment data. These sources of data are summarized in Table 1. The finfish species evaluated as representative important species (RIS) included bay anchovy, weakfish, striped bass, white perch, American shad, alewife, blueback herring, spot, and Atlantic croaker.

The availability of an extensive time series of in-plant, riverwide, and coastwide data allowed us to utilize empirical, retrospective approaches rather than relying only on the predictive, model-based approaches used in previous studies. Moreover, in performing modeling studies to supplement the retrospective approaches, we were able to use advanced methods and data sources that were unavailable for earlier Section 316(b) assessments at Salem.

## DEFINITION OF BENCHMARK OF AEI

We used three benchmarks of AEI — two relating to past operations, and one relating to current and future operations — to evaluate whether Salem operations may have caused or could cause AEI. For the first benchmark termed the “Balanced Indigenous Community” (BIC) benchmark, we evaluated whether the operation of Salem had upset or modified the balance of fish

**TABLE 1**
**Sampling Programs Used to Assess Impacts of Salem on Delaware Estuary Fish Populations and Communities**

| Program | Spatiotemporal Coverage | Gear (s) | Use in Impact Assessment |
|---|---|---|---|
| PSEG in-plant sampling and special studies | Salem cooling water system<br><br>1977-1998 | Various gears and collection methods | Estimation of entrainment and impingement loss rates; CMR estimates for stock jeopardy analysis |
| PSEG Nearfield Bottom Trawl Survey | RM 40-RM 61<br><br>1970-1982; 1986-1998 | 16 ft. otter trawl | BIC analysis; trends analysis |
| PSEG Baywide Survey | RM 0-RM 73<br><br>1979-1982, 1996-1998 | 16 ft. otter trawl<br><br>4ft. x 6ft. fixed frame pelagic trawl<br><br>1.6 ft. plankton net | CMR estimates for stock jeopardy analysis |
| DNREC Juvenile Trawl Survey | RM 6 – RM 59 (Delaware side only)<br><br>1980-1998 | 16 ft. otter trawl | trends analysis |
| NJDEP Beach Seine Survey | RM 59 – RM 140<br><br>1980-1998 | 100 ft. beach seine | trends analysis |
| PSEG White Perch Mark-Recapture Program | RM 50-119<br><br>1980-83, 1996-98 | 16 ft. otter trawl | CMR estimates for stock jeopardy analysis |
| New Jersey – Delaware American Shad Monitoring Program | Upper river<br><br>1975-1983, 1986, 1989, 1992, 1995, 1996 | Haul seine (marking), angler returns (recapture), hydroacoustic survey (1995-96) | CMR estimates for stock jeopardy analysis |
| ASMFC and NOAA stock assessments and coastal surveys | Atlantic coast and major estuaries | | Cumulative impact assessment |

species present in the Delaware Estuary, as reflected in species presence-absence data. For the second benchmark, termed "Continuing Decline in Abundance of Aquatic Species" (Trends), we evaluated trends in the abundance of age 0 fish belonging to the nine RIS, using one or more of the three available long-term trends data sets available for the estuary. For the third benchmark, termed the "Stock Jeopardy" benchmark, we used widely recognized models and fishery management reference points to evaluate potential current and future effects of Salem on the sustainability of the nine populations evaluated.

We evaluated all three benchmarks using a weight of evidence approach to determine the presence or absence of an AEI. It would be reasonable to conclude that Salem was not causing an adverse impact on the estuary if the fish community appeared to be in balance, if no species were exhibiting a long-term decline that could be attributable to Salem's operation, if stock assessment models indicated no jeopardy due to Salem's operations, and if the results from the three benchmarks were consistent with other data concerning the species in question.

## THE BALANCED INDIGENOUS COMMUNITY ANALYSIS

The purpose of the BIC analysis was to determine whether the fish community of the Delaware Estuary has changed during the period of operation of Salem in a way that might indicate the presence of an AEI. Ecologists use the term "community" to denote the entire assemblage of species present in a given location or habitat. Data on the number and relative abundance of species present in different communities are used to draw inferences concerning their evolutionary history, successional status, temporal stability, or degree of disturbance. Communities are said to be "diverse" if many species are present. Some ecologists[4,5] have argued that disturbances — including physical disturbances, pollution, and overharvesting — generally cause reductions in the diversity of communities. Although the general validity of this proposition has been questioned[6], empirical observations have demonstrated that the diversity of many types of biological communities is, in fact, reduced by a wide variety of environmental stresses[5]. For this reason, measures of species diversity are still used as indicators of the influence of environmental stress on biological communities.

Many indices of fish species composition have been proposed and used by ecologists[6,7]. As noted by Gotelli and Graves[6], most of these indices are highly correlated with one another. Moreover, many indices lack valid statistical tests and biologically meaningful interpretations. Hurlburt's[8] measures of species richness, defined as "…the number of species present, without any particular regard for the exact area or number of individuals examined," do not suffer from these defects. Hurlburt[8] distinguished two types of species richness measures: *numerical richness,* meaning the number of species present in a collection containing a specified number of individuals, and *areal richness*, meaning the number of species present in a given area or volume of the environment. Areal richness is also termed "species density." Our analysis used both numerical richness and species density as measures of fish community structure in the Delaware Estuary.

PSEG's Nearfield Bottom Trawl Program (Table 1) provides the only data set for which both prestartup and poststartup data are available; therefore, this data set was the only data set used for the BIC analysis. The bottom trawl samples the benthic stratum of the estuary, and it could be argued that this gear samples only part of the fish community present at any given location. However, due in part to the turbulence and high turbidity of the estuary in the vicinity of Salem, both pelagic and benthic fish species are represented in the bottom trawl collections. Moreover, since the BIC analysis is based on counts of the number of species present in a sample, irrespective of their relative abundance, the results should be relatively insensitive to gear-related sampling biases.

Unit 1 began commercial operation in 1978. Although preoperational testing was conducted prior to 1978, all of the years 1970 through 1977 are considered to be preoperational years for the purpose of the BIC analysis. If station operations were adversely affecting the fish community of the Delaware Estuary, it is unlikely that all possible effects would occur immediately. Moreover, for long-lived, slow-maturing species, several years would be required before mortality imposed on early life stages could result in reduced abundance of older fish. The years 1978 through 1985 are considered to be a "transitional period" for the purpose of the BIC analysis. The years 1986 through the present are considered to be the "operational period" for the purpose of the BIC analysis. The impact of station operations on the fish community of the Delaware Estuary was evaluated by comparing species richness and species density, as determined from the near-field bottom trawl data, between the 1970–1977 preoperational period and the post-1985 operational period.

A standard collection size of 650 was selected for the numerical richness calculations. Because the finfish species composition of the estuary changes seasonally, separate analyses were performed for the spring (April–May), summer (June–August), and fall (September–October) seasons. Fig. 2a shows the results for the summer season. The results show no apparent trend in species richness over time. Statistical tests performed using Heck's[9] method for calculating the variance in species richness estimates confirm that there is no statistically significant difference in species richness between the preoperational and the operational periods. Preoperational vs. operational differences in richness for the spring and fall seasons (not shown) are also statistically insignificant.

Figure 2b shows trends in species density for the summer season in preoperational, transitional, and operational periods. Fig. 2b shows an apparent increase in species density over time, from a mean of 3.98 species per sample in the preoperational period to a mean of approximately 4.82 in the operational period. Results for the spring and fall seasons are similar. For all three seasons, a two-sided t-test shows that the mean number of species per sample has been significantly higher in the operational period than in the preoperational period.

## THE TRENDS ANALYSIS

We reviewed all available fish monitoring data sets for the Delaware Estuary to determine their appropriateness for analyzing long-term abundance trends. The available data sets included the New Jersey Department of Environmental Protection (NJDEP) Beach Seine Survey, the Delaware Department of Natural Resources and Environmental Conservation (DNREC) Juvenile Trawl Survey, the DNREC Large Trawl Survey, the PSEG Nearfield Bottom Trawl Survey, and the PSEG Beach Seine Survey. The PSEG Beach Seine Survey was excluded because this survey began only in 1995. The DNREC Large Trawl Survey was excluded because of the numerous changes in sampling gear, locations of sampling, and months of sampling that have occurred since the program began in 1966. The PSEG Bottom Trawl Survey has been conducted using the same gear, and in the same locations and months since 1970; however, because of changes in gear deployment methods that affect sampling efficiency (use of a fixed-length towline prior to 1979; change in tow direction beginning in 1996), only data for the years 1979 through 1995 were considered sufficiently comparable to be used in the trends analysis. The DNREC Juvenile Trawl Survey has maintained consistent sampling methods since 1980; therefore, this data set was selected for analysis. The NJDEP Beach Seine Survey has been conducted since 1980; however, prior to 1986, the months and locations of sampling varied among years. Since 1986 sampling has been consistent from July through October, from RM 60 (RKM 96) to RM 140 (RKM 224); therefore, only the data from 1986 onward were used in the trends analysis. The estuary was divided into six sampling regions, and the data for each of the selected surveys were sorted by region and month. For the DNREC and PSEG surveys, species-specific age-length keys were used to select age 0 fish for analysis. The NJDEP data do not include lengths for species other than striped bass; therefore, for all other species, trends results for this data set reflect all ages present in each collection.

We constructed trends indices for each RIS by (1) determining the regions and months within which young-of-the-year fish of that species are found, (2)

(a)

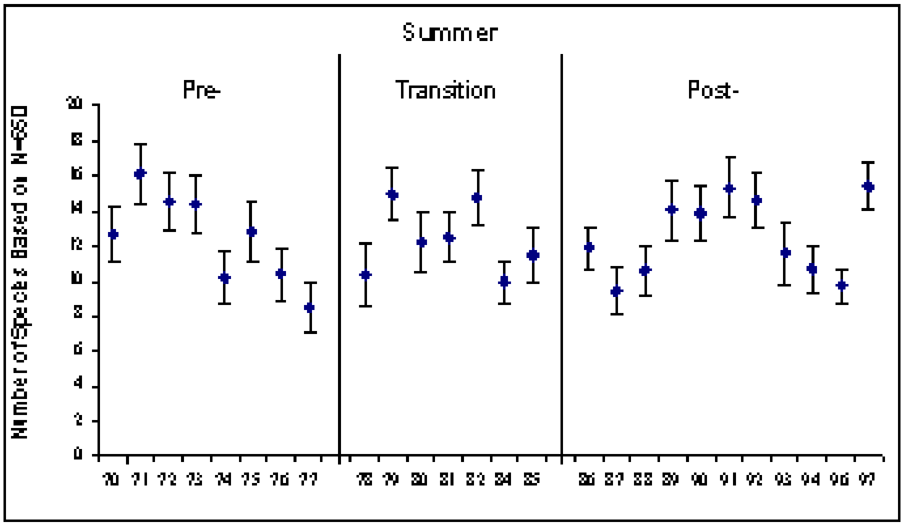

(b)

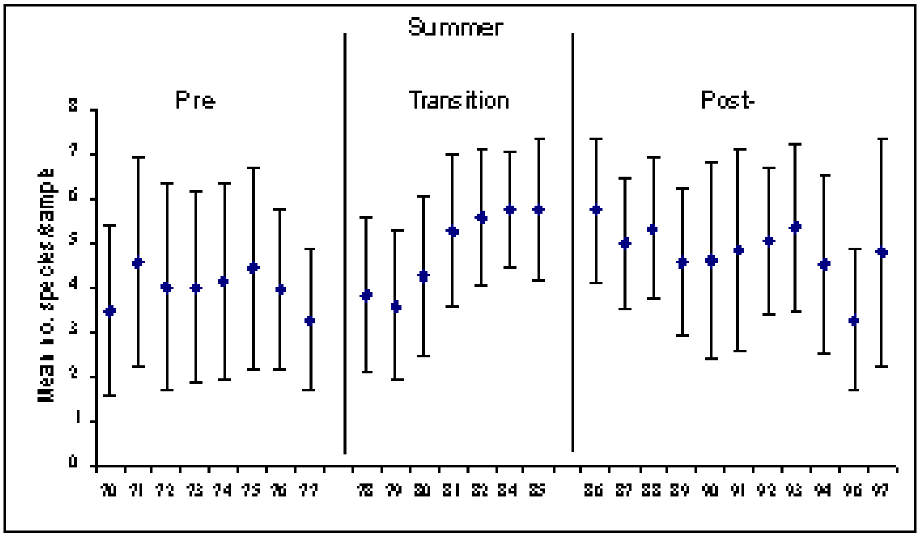

**FIGURE 2.** Species richness (a) and species density (b) in the vicinity of Salem during preoperational, transitional, and operational years.

calculating the average catch per haul (CPH) for each of the selected regions and months, and then (3) calculating an average CPH over all regions and months. Index values were calculated for a given data set only if data were available for all of the selected months and regions. We evaluated each data set separately, so that up to three independent trends analyses could be performed for each species.

We used two types of statistical analyses for detecting trends from the relative abundance indices, depending on the nature of the data from the sampling program. If sampling had been discontinuous (i.e., a break of one or more years in the time series for a given data set), then a test for differences in average CPH before and after the break was used. If sampling had been continuous over the entire data set, then a linear regression was used to test for a slope significantly different from 0. In addition to the statistical tests, we summarized the changes in abundance as percent change in the population per year.

Table 2 presents the percent change in abundance per year of age 0 fish (for the NJDEP survey, all fish collected) in the Delaware Estuary by species and program. Changes that are statistically significant are shown in boldface. Table 2 shows that most of the RIS have increased in abundance over the period covered in the trends analysis. For alewife, Atlantic croaker, striped bass, weakfish, and white perch, all three programs indicate an increase. Only the NJDEP Beach Seine Program samples American shad in adequate numbers; the NJDEP American shad index shows a statistically significant upward trend. Two of the three indices for bay anchovy indicate an increase in abundance; only the PSEG bottom trawl survey indicates a decrease.

Decreased abundance is indicated only for blueback herring and spot. The decline in abundance of blueback herring in the Delaware Estuary parallels a coastwide decline in the abundance of this species that began prior to the startup of Salem in 1977[10]. New Jersey and Delaware are at the northern end of the range of spot and Atlantic croaker; the abundances of both species in New Jersey and Delaware fluctuate with changing oceanic conditions. The abundances of these two species as demonstrated in both the Delaware Estuary surveys and the New Jersey and Delaware commercial landings appear to be inversely related (high abundance of one species is generally associated with low abundance of the other); the reason for this pattern is unknown.

## THE STOCK JEOPARDY ANALYSIS

We used population-level assessment models that extend the approaches used in previous assessments, drawing on recent advances in fisheries science and

**TABLE 2**
**Percent Change in Abundance per Year of Age-0 Fish (DNREC and PSEG Programs) or of All Ages Collected (NJDEP Program) for Each RIS Finfish Species**

| **Species** | **Program** | | |
|---|---|---|---|
| | DNREC Juvenile Trawl | NJDEP Beach Seine | PSEG Nearfield Bottom Trawl |
| Alewife | **55.4**[a] | 2.1 | 38.7 |
| American shad | NI[b] | **7.3** | NI |
| Atlantic croaker | +[c] | + | **3610.3** |
| Bay anchovy | 1.3 | **24.4** | **-4.8** |
| Blueback herring | **-5.5** | **-7.6** | NI |
| Spot | **-2.4** | **-8.1** | NI |
| Striped bass | **40.4** | **5.3** | NS[d] |
| Weakfish | **18.7** | 28.6 | 0.1 |
| White perch | **91.4** | **12.6** | **41.7** |

[a] Boldface indicates statistically significant change ($p < 0.05$).

[b] NI indicates that no index of abundance was calculated because of insufficient abundance in the regions sampled by a program.

[c] + indicates an increasing trend; percent change could not be calculated because the predicted initial population size from the regression model was zero.

[d] NS indicates no significant trend; percent change could not be calculated because the predicted initial population size from the regression model was zero.

management practice. Previous assessments used the conditional mortality rate (CMR) as a measure of impacts of power plants on fish populations[11,12,13]. The CMR is a rate of “fishing” mortality that is closely related to the instantaneous fishing mortality rate ($F$) used to quantify effects of fishing:

$$CMR = 1 - e^{-F_P} \tag{1}$$

where $F_P$ = instantaneous rate of mortality due to entrainment or impingement at a power plant.

Boreman and Goodyear[12] and Barnthouse and Van Winkle[13] discuss procedures for calculating CMRs for entrainment and impingement. The CMR does not account for compensatory mechanisms that can offset entrainment or impingement mortality, and the CMR cannot be used to project long-term effects of entrainment and impingement on fish populations. The term "compensatory mechanisms" refers to biological processes (e.g., competition for limited food resources or habitat) that reduce the growth rates of large populations and increase the growth rates of small populations.

Because of this limitation, we chose methods that use the CMR as an input to more advanced assessment approaches: the spawning stock biomass per recruit (SSBPR) approach and the spawner-recruit (S-R) approach. The SSBPR approach estimates the lifetime reproductive output of a recruit (usually defined as a 1-year-old fish), accounting for the expected reproduction of the fish at each future age and the probability that the fish will survive to reach that age[14]. When a population is subjected to mortality caused by fishing, the reproductive output of a typical recruit is decreased, because the probability of each recruit surviving to reproduce is decreased (in the case of fishing). For a population subjected to mortality due to cooling-water withdrawals, the probability of a spawned egg surviving to age 1 is decreased. This mortality is equivalent to removing the reproductive output that would have produced the lost recruits. To offset the losses and sustain the population, the survival or reproduction of the remaining fish must increase. Fisheries scientists have found that many fish populations continue to sustain themselves when fishing has reduced SSBPR to as low as 20% of the level found in an unfished population[15]. Information needed to estimate a biological threshold below which SSBPR should not be reduced was unavailable for most of the species evaluated in our assessment. For this assessment, we assumed that an AEI could not occur as long as SSBPR remained above 30% of the unfished value. We then estimated, for all species for which data sufficient to calculate a CMR were available (all RIS species except striped bass and Atlantic croaker), the SSBPR in the presence of the combined effects of Salem and fishing and compared that SSBPR to a reference point of 30% of the unfished SSBPR.

Fisheries managers also use the total spawning stock biomass (SSB) of a fish population as a measure of population status. The SSBPR approach does not explicitly calculate the influence of reduced egg production on future recruitment or spawning stock biomass (SSB). Any such calculation requires an S-R model, which expresses the relationship between the size of the spawning stock in a given year — measured in terms of total biomass — and the number of recruits that will be produced by that stock. To estimate the effect of Salem on SSB for each of the modeled fish populations, we employed an S-R modeling approach termed the "equilibrium spawner-recruit analysis" (ES-RA). The ES-RA model extends the SSBPR approach by considering: (1) uncertainty concerning the values of critical life history parameters and (2) the relationship between SSB and recruitment. Since the ES-RA model includes more information than the SSBPR approach and should involve a lower degree of uncertainty, we used a less conservative reference point of 20% of the unfished value for the SSB analysis.

The ES-RA model requires the same data used in the SSBPR approach, i.e., age-specific estimates of natural mortality, fishing/power plant mortality, and weight or fecundity. In addition, the ES-RA requires an estimate of the slope of the spawner-recruit curve at the origin, i.e., the number of recruits produced by each spawner at very low population sizes. This slope is measured by the $\alpha$ parameter of the Ricker[16] or Beverton-Holt[17] spawner-recruit models.

Ideally, estimates of $\alpha$ would be obtained for each population of interest from the analysis of observations of spawner and recruit abundance for that population. However, such data are rarely available for species that are vulnerable to entrainment and impingement. Recently, Myers and Mertz[18] demonstrated that a statistical approach termed "meta-analysis" can be used to estimate $\alpha$ for a population of interest from estimates of $\alpha$ derived from long-term spawner recruit data for other populations.

Meta-analysis was used to derive estimates of $\alpha$ for use in the ES-RA analysis. Myers et al.[19] have compiled S-R data for more than 600 fish populations. Of the 600 data sets, 246 were considered sufficiently complete to provide reliable estimates of $\alpha$. Of these 246 fish populations used, 109 were anadromous, 11 were freshwater, and 126 were marine or estuarine. The data set included 57 species belonging to 21 families and 8 orders.

Distributions of the parameter $\alpha$ were generated from the available S-R data using the methods described by Myers et al.[18,20]. Although either the Ricker or the Beverton-Holt models could have been used to estimate $\alpha$ from the S-R data sets, the Ricker model was chosen because it provided more conservative (precautionary) fits than the Beverton-Holt; i.e., on average it provided lower estimates of $\alpha$ for each species. Two approaches were employed in the choice of data sets to be used in the meta-analysis for each of the individual RIS. The first approach was to select closely related species, e.g., species within the same genus as the individual RIS. This approach was used for the alewife, American shad, and blueback herring. The second approach was to select species with similar life history characteristics and environmental tolerances, e.g., with the same type of reproduction (i.e., oviparous vs. ovoviviparous), habitat (i.e., anadromous, freshwater, or marine), natural mortality rates, longevity, fecundity, age at maturity, latitudinal distribution, and ambient temperature. This approach was used for all other species evaluated.

Given the above parameters, the ES-RA model was used to calculate equilibrium SSB for each species as a function of plant and fishing mortality. The Beverton-Holt model was used for this step in the analysis because it is more precautionary than the Ricker model. Specifically, for any given level of entrainment or impingement mortality, the Beverton-Holt model predicts a greater reduction in equilibrium spawner abundance than does the Ricker model. Assuming a Beverton-Holt type S-R relationship, the equilibrium biomass and yield equations derived by Lawson and Hilborn[21] were modified to include entrainment and impingement. The model assumes a life history in which power plant impacts are divided into two phases: a "precompensation" phase, and a "postcompensation" phase. Precompensatory mortality due to entrainment or impingement can be partly or largely offset by subsequent density-dependent mortality, resulting in a much smaller impact on age 1 abundance than would be predicted by a model that assumes no compensation (e.g., the CMR model). Postcompensatory mortality cannot be offset by compensation and translates directly into proportional reductions in age 1 abundance and subsequent recruitment to the adult population. Compensation in this case is delayed until the subsequent generation of fish, in which age 0 survival increases as a result of reduced adult abundance. Fishing mortality, according to this model, is always postcompensatory mortality.

The meta-analysis produced probability distributions of $\alpha$ for each fish species; these distributions were incorporated in the ES-RA calculations using Monte Carlo analysis. Uncertainty concerning the actual values of other key parameters, such as natural mortality rates, fishing mortality rates, and the fraction of the population present in the Delaware Estuary, was also addressed using Monte Carlo analysis. Probability distributions for these parameters were derived from the scientific literature or from best professional judgment concerning the possible range of values.

The results of the ES-RA calculations were expressed as ratios of spawning stock biomass, including the combined effects of fishing and power plants, to the spawning biomass of an unexploited population ($SSB_0$). CMRs were available for weakfish, bay anchovy, spot, white perch, alewife, American shad, and blueback herring; the ES-RA model was applied to each of these species. To demonstrate the approach, the discussion below focuses on weakfish.

Figure 3 presents results of the stock jeopardy analysis for weakfish. Life history parameters used in the SSBPR and ES-RA calculations are presented in Table 3. These parameters were taken from the most recent stock assessment available at the time this assessment was performed[22].

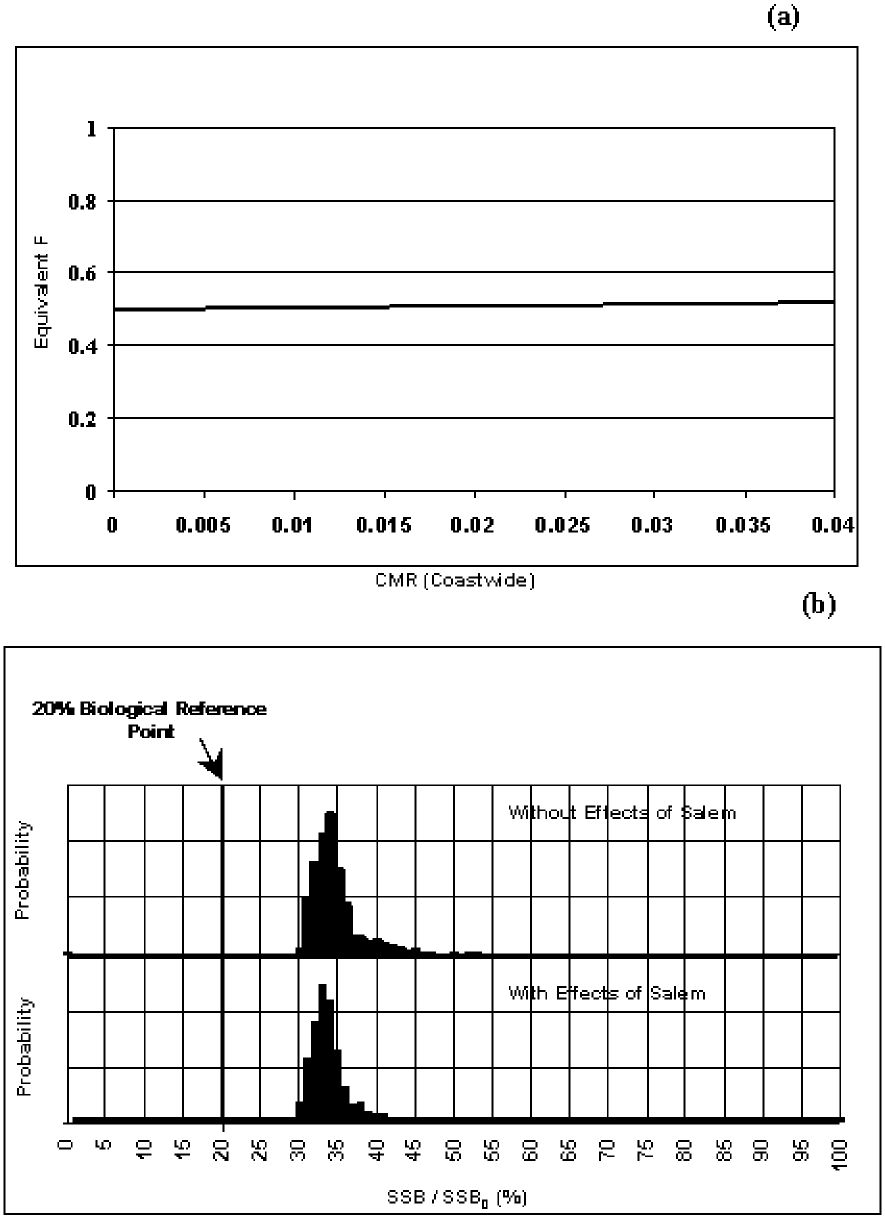

**FIGURE 3.** Results of stock jeopardy analysis for weakfish. Fig. 3(a) shows the influence of Salem on weakfish SSBPR, expressed as an incremental addition to the target rate of fishing for weakfish, for a range of assumptions concerning the coastwide CMR due to Salem. Fig. 3(b) shows the influence of Salem + fishing on weakfish SSB, expressed as a fraction of the equilibrium SSB for an unfished population.

**TABLE 3**
**Life History Parameters for Weakfish[22]**

| Age | M | Vulnerability to Fishery | % Female | % Mature | Fecundity | Weight (lbs.) |
|---|---|---|---|---|---|---|
| 1 | 0.25 | 10% | 50% | 30% | 6824 | 0.26 |
| 2 | 0.25 | 50% | 50% | 85% | 32973 | 0.68 |
| 3 | 0.25 | 100% | 50% | 90% | 71387 | 1.12 |
| 4 | 0.25 | 100% | 50% | 100% | 130848 | 1.79 |
| 5 | 0.25 | 100% | 50% | 100% | 272716 | 2.91 |
| 6 | 0.25 | 100% | 50% | 100% | 1041839 | 6.21 |
| 7 | 0.25 | 100% | 50% | 100% | 1454325 | 7.14 |
| 8 | 0.25 | 100% | 50% | 100% | 2147645 | 9.16 |
| 9 | 0.25 | 100% | 50% | 100% | 2778770 | 10.83 |
| 10+ | 0.25 | 100% | 50% | 100% | 3547138 | 12.50 |

Weakfish are managed as a mixed coastwide population ranging from Nova Scotia to Florida, although recent evidence[23] suggests that there is substantial fidelity to natal estuaries. We assumed that the weakfish population from North Carolina to Massachusetts constitutes a distinct breeding population. Based on analysis of state landings data, we assumed that the Delaware Estuary contributes 10–20% of the coastal stock. A reliable estimate of the rate of fishing mortality for weakfish was unavailable when this assessment was performed[22] However, a target fishing rate ($F$) of $F = 0.5$ had been established by the Atlantic States Marine Fisheries Commission (ASMFC). We used the SSBPR model to evaluate the potential impact of Salem in the context of this target fishing rate. Combining mortality due to Salem with fishing mortality is, in terms of impact on SSBPR, equivalent to incrementally increasing the rate of fishing on the adult stock. Fig. 3a shows the relationship between the coastwide CMR and the equivalent fishing rate for weakfish. Assuming that the Delaware Estuary contributes 20% of the coastwide weakfish population, the estimated coastwide CMR (0.034) is equivalent to raising the fishing rate for weakfish from the target rate of 0.5 to 0.517.

Fig. 3b shows the influence of fishing and Salem on total SSB for weakfish, including the stock-recruitment relationship estimated using the ES-RA. Because neither empirical estimates of $F$ nor an approved S-R model for weakfish were available at the time this assessment was performed, we assumed that the weakfish population would in the future be fished at a rate close to the MSY level computed from the ES-RA model (approximately 35% of the unfished SSB). Fig. 3b shows that, even including uncertainty in key parameters, SSB is predicted to remain close to the MSY level and well above the overfishing reference point (20% SSB).

Results of the stock jeopardy analyses for other species showed even smaller potential impacts than were found for weakfish. In no cases did predicted impacts on SSBPR or SSB approach or exceed the overfishing reference points.

## CONSISTENCY WITH OTHER INFORMATION CONCERNING POPULATION STATUS

The results of our analyses are consistent with other information concerning the status and trends of the Delaware Estuary's biological resources, and together they indicate that the operation of Salem over the past 20 years has not had an adverse impact on those resources.

Water quality in the Delaware River has improved dramatically since 1970. The Delaware Estuary's watershed contains one of the heaviest concentrations of industrial facilities in the world[24]. Until the 1950s, most of the wastewater generated in the watershed was discharged to the estuary without primary treatment, and secondary treatment only became prevalent in the 1980s[25]. Water quality was especially poor in the vicinity of Philadelphia, where dissolved oxygen concentrations frequently fell to zero[25]. Low DO blocked the passage of migratory fish such as American shad and other alosids, and destroyed or degraded much of the spawning and nursery habitat utilized by striped bass[26]. Since the mid-1980s, DO levels in the vicinity of Philadelphia have greatly improved. High densities of striped bass ichthyoplankton were observed in 1988, in a region of the river where ichthyoplankton had been absent in 1970[27]. Striped bass juvenile density, according to both the DNREC and NJDEP monitoring programs, was near zero during the early 1980s but increased rapidly thereafter. Weisberg et al.[28] attributed increases in the abundance of striped bass, white perch, and American shad in the Delaware Estuary to improvements in water quality.

Fisheries data are also consistent with the results discussed above. In response to restrictions placed on commercial and recreational fishing, spawning stock biomass of both striped bass and weakfish have risen to the highest levels observed over the past 20 years[29,30]. Coastal landings of American shad and river herring (alewife + blueback herring) have declined steadily throughout this century, in part due to overfishing but also due to declining water quality and blockage of tributaries by dams[10,31]. Within the Delaware River, however, both annual recruitment and spawning stock size are increasing[32]. No recent stock assessments are available for river herring. Stock assessments are also unavailable for spot and Atlantic croaker; however, landings data indicate that the abundance of both species fluctuates greatly in New Jersey and Delaware waters[33]. These fluctuations are probably related to fluctuations in coastal water temperatures that expand and contract the ranges occupied by these species. Even in peak years, catches in New Jersey and Delaware comprise no more than a few percent of total coastwide landings[33]. Available data for white perch in the Delaware Estuary show that commercial landings have increased since the mid-1980s, coincident with observed improvements in water quality.

It could be argued that improvements in water quality and reductions in fishing effort are simply confounding influences that mask adverse effects due to Salem, and therefore that data demonstrating that populations and communities are healthy are irrelevant to determining the presence or absence of adverse impacts. Perhaps the observed increases would have been even greater if not for entrainment and impingement at Salem. Arguments of this type cannot be logically disproved. They can, however, be evaluated qualitatively using the “risk hypothesis” approach discussed in EPA’s Guidelines for Ecological Risk Assessment[34], as discussed below.

Of the finfish species we evaluated, the species that appear to be the most vulnerable to Salem include bay anchovy, weakfish, striped bass, and white perch. If entrainment and impingement were depleting vulnerable populations, then the abundance of one or more of these populations should decline. If the depleted populations were prey species such as bay anchovy — by far the most abundant prey species in the Delaware Estuary — then the abundance of predator species such as weakfish, striped bass, and white perch might also be expected to decline. If, on the other hand, the depleted populations were predators such as weakfish, striped bass, and white perch, then the abundance of prey species such as bay anchovy might be expected to increase. These types of changes have not occurred during the 20-year operational history of Salem. Instead, the abundances of all of the above species has increased.

The changes that have been observed over the last 20 years are inconsistent with the expected effects if Salem had been having an AEI on fish populations, but are consistent with the expected effects of water-quality improvements and reduced fishing effort. Species that utilize areas of the estuary that were formerly affected by poor water quality, including striped bass, white perch, American shad, and alewife, experienced substantial population growth when water quality improved in the 1980s. Species such as striped bass, weakfish, and American shad, for which harvests were restricted to promote recovery of depleted populations, increased in abundance following the enactment of those restrictions. Any impacts due to Salem clearly were too small to affect the responses of these populations to management changes intended to improve the Delaware Estuary and its biological resources.

## CONCLUSIONS

Investigation of responses of fish populations to entrainment and impingement should involve evaluation of multiple lines of evidence, including both empirical observations and model-derived predictions. For the 1999 Salem Section 316(b) Demonstration, we developed three independent benchmarks of AEI and used multiple sources of data and modeling approaches to evaluate each benchmark. All three analyses indicated that the fish populations and communities of the Delaware Estuary are healthy and show no evidence of an adverse impact due to Salem, at least as defined in terms of changes in community balance or reduced sustainability of representative populations. Although conclusions derived regarding each individual benchmark are subject to multiple uncertainties, the concordance of all of our analyses appears sufficient to demonstrate that the influence of Salem is small compared to the influence of other major factors that affect the estuary.

The exact form of our analysis was constrained by the types of data available; the approach would have to be modified for application to other power plants and ecosystems. However, the general principal of evaluating multiple benchmarks, within a clearly defined analytical framework, should be applicable to all Section 316(b) determinations.

## ACKNOWLEDGEMENTS

The authors acknowledge the support of the John Balletto, Maureen Vaskis, and other members of the PSEG staff for the support and encouragement they provided during the conduct of this assessment. We also gratefully acknowledge the assistance of Jennifer Field, John Posey, John Seibel, and Lorraine Read, without whom the assessment would never have been completed.
